# Anti-MDA5+ dermatomyositis following SARS-COV-2 infections: a systematic review

**DOI:** 10.3389/fimmu.2025.1565803

**Published:** 2025-09-02

**Authors:** Simone Lattarulo, Francesca Centrone, Maria Chironna

**Affiliations:** ^1^ Department of Interdisciplinary Medicine-Hygiene Section, University of Bari, Bari, Italy; ^2^ Hygiene Unit, Policlinico University Hospital, Bari, Italy

**Keywords:** SARS-CoV-2, MDA5+ DM, autoantibodies, type-I interferon signature, COVID-19

## Abstract

**Background:**

Anti-MDA5+ dermatomyositis (DM), also called anti-MDA5+ syndrome, or clinically amyopathic dermatomyositis (CADM), is characterized by extra-muscular DM manifestations such as skin rash, arthralgia, and rapid progressive-interstitial lung disease. Between 2020 and 2024, an increase in serum titer of anti-MDA5 autoantibodies (AABs) and MDA5+ DM cases was registered among the general population. Given the role of MDA5 as a viral-RNA sensor, it is considered a key molecule in rheumatological disorders, as studies show its activity is triggered by viral infection. Here, we conducted a systematic review of studies reporting an unambiguous temporal link between SARS-CoV-2 infections and development of MDA5+ DM. The aim was to clarify our understanding of this idiopathic rheumatic nature.

**Methods:**

This review meets Preferred Reporting Items for Systematic reviews and Meta-Analyses guidelines (PRISMA). The Google Scholar, PubMed, Scopus and ScienceDirect were searched using appropriate keywords to identify relevant studies published from 2020–2025. Twenty-nine studies concerning the development of MDA5+ DM in COVID-19 patients, as well as molecular pathogenetic mechanisms and pharmaceutical treatments were included.

**Results:**

Anti-MDA5 antibodies have been detected in patients with COVID-19, as well as in sera from post-COVID patients, and their presence correlates positively with disease severity. The onset of MDA5+ DM, in different phenotypic variants, increased during the COVID-19 pandemic, paralleled by an increase in the incidence of juvenile idiopathic inflammatory myopathies (JIIM). The literature here reported shows that MDA5+ DM arises after primary SARS-CoV-2 infection, which could stimulate an antiviral pathway overactivation, leading to innate and adaptive immune cells recruiting, cytokine storm, and synthesis of autoantibodies.

**Conclusion:**

This review provides evidence for a link between primary SARS-CoV-2 infections, anti-MDA5 AABs synthesis and emergence of MDA5+ DM in phenotypically different variants such as MIP-C, driven by the virus’s inclination to trigger type-I interferonopathy in genetically predisposed individuals.

**Systematic review registration:**

https://www.crd.york.ac.uk/prospero/, identifier 1129317.

## Introduction

Viruses can be a trigger for rheumatic diseases. In turn, patients with rheumatic diseases are more susceptible to viral infections due to immunosuppression caused by the illness itself and/or related pharmacologic treatment (1–5). The global pandemic caused by SARS-CoV-2 affected the onset of many autoimmune disorders (6), one of the most intriguing being anti-MDA5+ dermatomyositis (DM). Although anti-MDA5+ DM is classified in the group of idiopathic inflammatory myopathies (IIM), which typically involve muscle tissue, it predominantly affects the skin with hypomyopathic or no muscle involvement and, more rarely, internal organs such as the lungs, with rapidly progressive interstitial lung disease (RP-ILD) (7, 8). Anti-MDA5+ DM is characterized by autoantibodies (AABs) directed against melanoma differentiation-associated gene 5 (MDA5), which were recognized in 2009 by Sato et al. (9). Before that time, the anti-MDA5+ DM was classified by dermatologists as clinically-amyopathic dermatomyositis (CADM) (7), but according to the latest Dermatomyositis Classification it has been recently termed MDA5+ DM (8, 10). The clinical manifestations of disease can be clustered in three main phenotypic subgroups, each one with a different prognosis: anti-MDA5+ rheumatic DM (skin lesions with arthralgia/arthritis) with good prognosis; anti-MDA5+ vasculopathic DM (skin vasculopathy and Raynaud’s phenomenon) with intermediate prognosis; anti-MDA5+ RP-ILD DM, with a prevalence from 50 to 100% and the poorest prognosis and high early mortality (8, 11). This evidence suggests that MDA5+ DM is mostly a systemic syndrome, rather than a musculocutaneous disease, thereby it has also been named “anti-MDA5+ syndrome”. Moreover, because many patients result amyopathic, the myositis-specific antibody (MSA) classification seems to be inappropriate (11). RP-ILD, pneumomediastinum and ground-glass opacities are prominent characteristics of both anti-MDA5+ dermatomyositis and COVID-19, in addition to vasculopathy, thrombosis, fever, hyperferritinemia, and cytokine storm syndrome. This remarkable similarity has prompted global experts to investigate COVID-19 as a potential human model for anti-MDA5+ DM (12). In fact, although the etiological factors of MDA5+ DM are unknown, there is growing evidence on viral infections as an environmental trigger for such rheumatic conditions, and on a seasonal pattern in condition onset between October and March (13, 14). Dermatomyositis affects women two to three times more than men, particularly Asian and African American women, with a bimodal age range at diagnosis: children aged 5–14 years and adults aged 40–60 years (15). Caucasian adult populations account for 7-16% of MDA5+ DM cases, while Asian populations for 11-60% (16). MDA5 is a crucial antiviral factor reportedly involved in SARS-CoV, MERS-CoV and SARS-CoV-2 (13). It is a cytoplasmic receptor encoded by the *IFIH1* gene, which binds to viral dsRNA, thereby triggering synthesis of type-I–III interferons, inhibiting viral replication and activating innate immune responses (17). Remarkably, MDA5 is also involved in autoimmune disorders such as MDA5+ DM, underlying a common pathophysiological mechanism between COVID-19 and MDA5+ DM (17, 18). These data, along with an increase of rheumatic “post-COVID” symptoms, suggest that a high anti-MDA5 AABs titer is related to an uncontrolled inflammation and autoimmune response following SARS-CoV-2 infection, promoting tissue damage with some long-term sequelae named post-COVID syndrome (PCS) in genetically predisposed patients (18–20). Given the lack of a certain association, the aim of this review is to bring together the evidence (published from 2020–2025) suggesting that exposure to SARS-CoV-2 precedes development of MDA5+ DM, to put forward a model for disease development, and to further increase our understanding of this idiopathic rheumatic condition.

## Methods

### Literature search strategy

**Figure 1:**
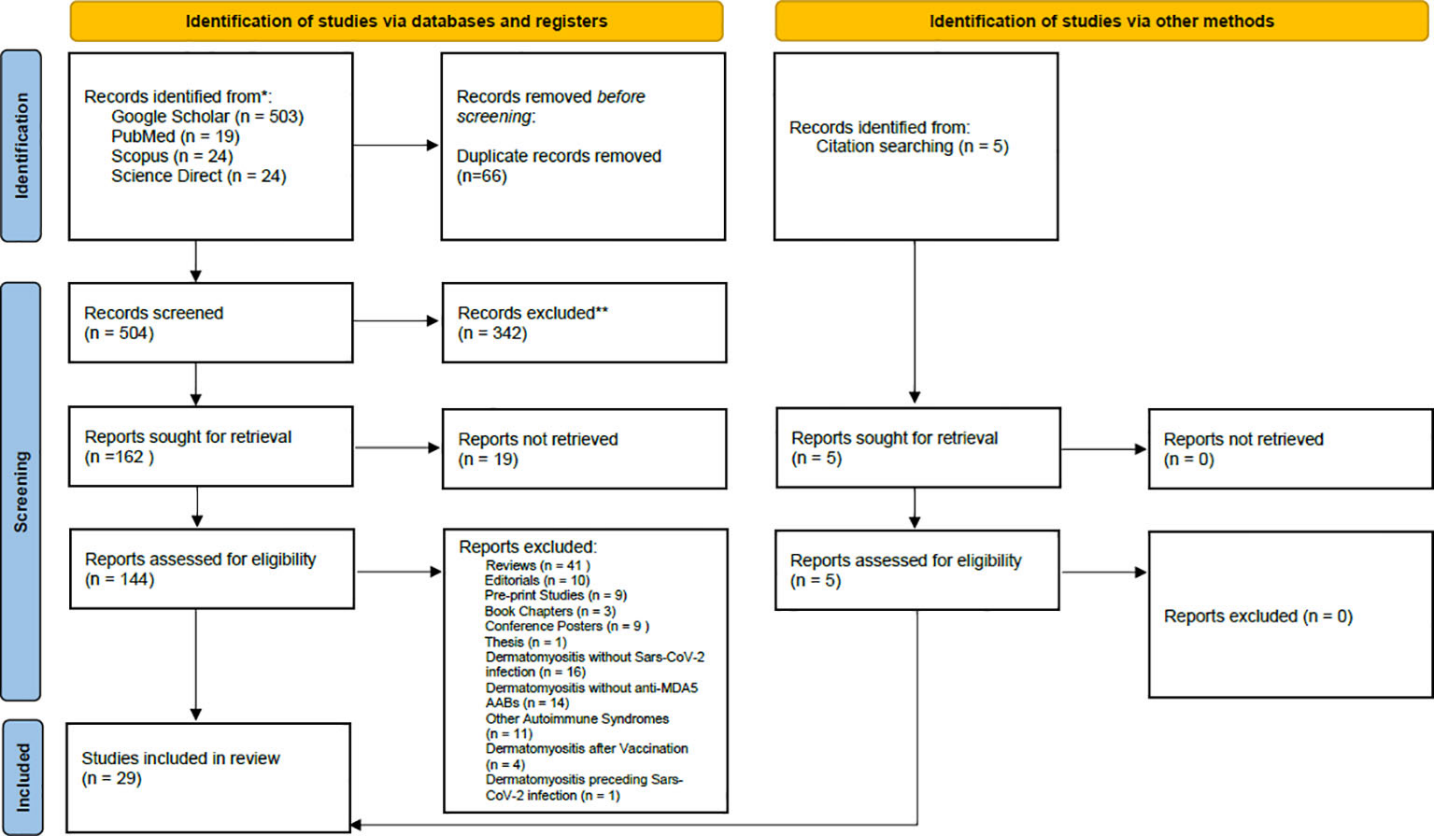
PRISMA flow chart of search, inclusion and exclusion screening, and accepted studies of the review about Dermatomyositis Anti – MDA5 (AMD) developed after Sars-CoV-2 infection.

The review was conducted according to Preferred Reporting Items for Systematic reviews and Meta-Analyses (PRISMA) guidelines (**Figure 1**). The PubMed, Google Scholar, Scopus and Science Direct databases were searched to identify relevant studies published from 2020 to 2025 using the following keywords with the AND Boolean Operator: “(SARS-CoV-2) AND (Dermatomyositis) AND (anti-MDA5+)” AND (COVID-19).

### Study selection

The review focuses on MDA5+ DM development during/after SARS-CoV-2 infection, and on molecular studies reporting increases in serum anti-MDA5 antibody titer consequent to interferonopathy caused by RNA virus infection (e.g., SARS-CoV-2). Articles were considered eligible if they met the following inclusion criteria: case reports or research articles written in English, with a focus on MDA5+ DM developing after SARS-CoV-2 infection. Book chapters, reviews, pre-print works, conference abstracts, and practice guidelines were excluded. The Rayyan^©^ automation tool (21), which removes all duplicates and helps full-text assessment, was used to screen resources (**Figure 1**). Studies were excluded during the full-text assessment if they reported vaccine-induced MDA5+ DM, DM characterized by autoantibodies other than anti-MDA5, SARS-CoV-2 infection in patients who already developed DM, and MDA5+ DM with a pathogenesis not related to COVID-19.

## Results

### Studies retrieved

The initial research identified 570 articles. After removing duplicates and studies marked as ineligible according to the title and abstract, 144 articles were full text screened. Articles reporting development of MDA5+ DM after Covid-19 vaccination, cases of DM without anti-MDA5 antibodies, MDA5+ DM not associated with COVID-19 infection, and MDA5+ DM pre-existing before COVID-19 infection were excluded. 29 articles were included in the systematic review (**Figure 1**): nineteen research papers and ten case reports (**Table 1**), amongst which two additional research articles and other three case reports were identified by citation searching (**Figure 1**). Among the papers identified, four focus on the production of anti-MDA5 autoantibodies during or after SARS-CoV-2 infection (**Table 2**); five on the development and characteristics of MDA5+ DM (**Table 3**); nine (**Table 4**) on the possible pathogenic molecular mechanisms involved; one on the most effective drug treatments tested to date (**Table 5**). We wanted to include these four different sets to analyze all aspects common to COVID-19 and MDA5+ DM comprehensively and organically, and to explain the increased incidence of this autoimmune disease in relation to infection and not vaccination.

**Table 1:** Characteristics of MDA5+ DM patients.

Case Reports	Nationality/Ethnicity	Gender	Age (years)	Ferritin values	CK values	Hemoglobin values	CRP values	Anti-MDA5 values	Symptomatology	SARS-CoV-2 infection	Vaccination
Keshtkarjahdromi M. et al., 2021 (48)	Caucasian	female	65	> 16,500 ng/mL	441 units/L	10.1 gm/dL	67 mg/L	High positive	Erythematous rash, heliotrope rash, Gottron’s sign, tachycardia, tachypnea., Macrophage Activation Syndrome (MAS). History of psoriasis	2 months prior hospitalization	no data
Suparna M.Y. et al., 2022 (50)	Indian	Two females	Case 1: 66 Case 2: 45	–	71 U/l	–	–	Case 1: anti-MDA5 Positive (3+); Case 2: anti-SAE1 positivity only	Case 1: history of diabetes mellitus, heliotrope rash, gluteal cutaneous ulcerations Case 2: periorbital erythematous edema	Case 1: 3 months before hospitalization Case 2: no data	Case 1: no data Case 2: Covishield, second dose three weeks before the hospitalization
Juganaru E. et al., 2022 (49)	Romanian	Female	31	–	–	–	–	Positive	Arthralgia, Gottron’s sign, digital ulcerations, nailfold erythema, pulmonary nodules	Days before the hospitalization	no data
Anderle K. et al., 2022 (16)	Bangladeshi	male	20	3933 μg/L	246 U/L	–	0.6 mg/dL	14 U/mL	Polyarthralgia, fatigue, sore throat, Gottron’s sign, hyperkeratotic papules, pulmonary nodules, ARDS	2,5 months prior hospitalization	no data
Stepien B. et al., 2023 (51)	Poland	Female	57	392.3 μmol/l	–	–	–	Positive (++)	Lower limbs skin ulcerations, calcinosis cutis	Two years before	no data
Chun Man N.G. et al., 2023 (52)	Chinese	Female	55	2622 pmol/L	345 IU/L	–	–	Strong positive	Periorbital Edema, Polyarthralgia skin rash, weight loss, ILD	One month before the hospitalization	Three doses of mRNA vaccine (BioNTech)
Lau T. et al., 2024 (53)	–	Male	2	–	–	–	–	positive	Cough, fever, progressive weight loss, organizing pneumonia,	Before hospitalization	No data
Arai N. et al., 2024 (54)	Japanese	Male	73	–	–	–	–	positive	Dyspnea, ground-glass opacities in lungs, hematuria, renal dysfunction	Before hospitalization	No data
Puthuman R.M. et al., 2024 (56)	Latin America	Two females (cases 1 and 3) One male (case 2)	Case1: 54 Case 2: 69 Case 3: 70	–	–	–	–	Case 1: Positive Case 2: Positive Case 3: Positive	Case 1: skin rash, pruritus, dyspnea, ground glass opacities, ILD Case 2: ILD, lymphoid hyperplasia Case 3: Fever, chills, malaise, cough, ILD	Case 1: no data Case 2: no data Case 3: two months prior hospitalization	Case1: no data Case 2: no data Case 3: no data
Armstrong SA et al., 2025 (55)	–	Case 1: female Case 2: male	Case 1: 87 Case 2: 84	–	–	–	–	Case 1: positive (32) Case 2: positive (27) (normal <20)	Case 1: emphysema, ground-glass opacities, MDA5 SP-ILD Case 2: dyspnea, ground-glass opacities, MDA5 SP-ILD	Case 1: one year before presentation Case 2:	Case 1: no data Case 2: no data

**Table 2:** Characteristics and main findings of the studies regarding the increase of anti-MDA5 AABs during SARS-CoV-2 infection.

N	Author	Year	Journal	Country	Type of study	Sample size	Main findings
1	Wang G. et al. (18)	2020	Frontiers in Immunology	China	Retrospective	274	Anti-MDA5 was present in COVID-19 patients: high titers of AAB correlated with severity of disease
2	Liu Y. et al. (27)	2021	Journal of Translational Medicine	USA	Research article	6062	SARS-CoV-2 infection led to AAB responses with a sex-specific pattern, including anti-MDA5 antibodies
3	Visvabharathy L. et al. (25)	2024	ImmunoHorizons	USA	Research article	46	Patients affected by a mild primary COVID-19 infection developed neuro-PASC symptoms, with an increase in AABs, particularly anti-MDA5. Vaccination protected against autoimmunity.
4	Garcìa-Bravo L. et al. (24)	2024	MDPI Biomedicines	Spain	Retrospective	788	Anti-MDA5 was detected in 11,68% of patients who developed IIM after Sars-CoV-2 infection and/or vaccination

**Table 3:** Characteristics and main findings of the studies regarding increased MDA5+ DM incidence following COVID-19 pandemic and the pathophysiological mechanisms involved.

N	Author	Year	Journal	Country	Type of study	Sample size	Main findings
1	Matsuo T. et al. (31)	2022	Frontiers in Immunology	Japan	Research Article	63	Presence of anti-MDA5 is associated with reduced T wave amplitude in electrocardiographic trace. Such phenomenon could be triggered by viral infections like Sars-CoV-2.
2	You H. et al. (29)	2023	Rheumatology	China	Epidemiological study	272	The window risk for RP-ILD onset is comprised between 3–6 months. It is suggested that Sars-CoV-2 triggers it.
3	Perfetto J. et al. (32)	2023	Pediatric Rheumatology	USA	Retrospective cross-sectional study	51	Comparison of data pre- and post-COVID-19 pandemic; 10/51 pediatric patients developed JIIM after 2020.
4	Kaniecki T. et al. (28)	2024	The Journal of Rheumatology	USA	Observational Survey	46	44% of survey responders reported a history of previous Sars-CoV-2 infection; a quarter of these reported anti-MDA5 at serum quantification.
5	David P. et al. (19)	2024	eBiomedicine, The Lancet	UK	Retrospective observational study	60	Increase in MDA5-autoimmunity as MIP-C phenotype in the Yorkshire region (UK) during the COVID-19 pandemic (2020–2022).

**Table 4:** Characteristics and main findings of the studies regarding molecular causes of aberrant IFN-I activation.

N	Author	Year	Journal	Country	Type of study	Sample size	Main findings
1	Song B. et al. (35)	2021	Cell Press Immunity	China	Research Article	–	Activation of IFN-I and IFN-III pathways requires long polyubiquitin K63 chains to stabilize by tethering the interaction between MDA5 CARD domains and the MAVS on the mitochondrial membrane.
2	Gono T. et al. (20)	2022	Rheumatology	Japan	Research article	6	Macrophages activated by RNA virus infections induce a cytokine storm, which leads to ILD in MDA5+ DM.
3	Zhang Z. et al. (39)	2023	Frontiers in Immunology	China	Research article	131	Transcriptomic analysis revealed two subsets of genes in common between IIM and COVID-19. One was related to the MAPK pathway, the other to the IFN signaling.
4	Wang Y. et al. (38)	2023	Frontiers in Immunology	China	Research article	69	Transcriptomic profiling, serum profiling and statistical analysis of MDA5+ DM patients demonstrated a correlation between IFN-I signature and disease.
5	He J. et al. (41)	2023	Rheumatology	China	Research Article	10	scRNA-seq was applied to PBMCs from MDA5+DM patients, revealing type-I IFN genes overexpression after viral triggering.
6	Ichimura Y. et al. (40)	2024	PNAS Immunology and Inflammation	Japan	Research Article	-	Viral-mimicking infection in MDA5-immunized mouse model stimulates an upregulation of type-I interferons along with ILD.
7	Ghoreshi ZA et al. (37)	2024	American Journal of Tropical Medicine and Hygiene	Iran	Research Article	153	Expression of IFIH1 gene increases with increasing infection intensity.
8	Shi J. et al. (43)	2025	Clinical and Translational Medicine	China	Research article	5	scRNA-seq analyzed the transcriptomic asset between pulmonary cells and PBMCs. Inflammation asset was concentrated in lungs, consistently with peripheral lymphopenia. IFN-I overactivation was observed in PBMCs required in lungs, consistently with viral infections.
9	Zhang Q. et al. (36)	2025	Advanced Science	China	Research Article	–	USP8 dictates MDA5 protein homeostasis and is considered a potential target for autoimmune conditions treatment.

**Table 5:** Characteristics and main findings of the study regarding pharmacological treatment of COVID-19.

N	Author	Year	Journal	Country	Type of study	Sample size	Main findings
1	Ferro F. et al. (45)	2024	International Journal of Molecular Sciences	Italy	Research article	246	A rheumatological approach using Baricitinib to target Janus Kinase significantly reduced the mortality rate of COVID-19 patients in the ICU.

### Detection of increased anti-MDA5+ antibody levels during SARS-CoV-2 infection

IgG autoantibodies that develop during a SARS-CoV-2 infection target many autoantigens associated with rare disorders, such as myositis (22). Interestingly, patients with PCS have high levels of autoreactive naive B cells, which are known to be a source of autoantibodies (2, 4). The development of autoimmune conditions following COVID-19 infection could be associated with transient immunosuppression of innate and acquired immunity leading to loss of self-tolerance and inappropriate immune reconstitution in genetically susceptible individuals (2, 4, 23). The first study to detect anti-MDA5 AABs in COVID-19 patients (18) was retrospective and reported a positivity rate of 48.2%. COVID-19 patients who were positive for anti-MDA5 AABs tended to have a much longer disease course, a higher incidence of respiratory failure and shock, and dysfunction of other organs. Another retrospective study (24) analyzed the profile of myositis autoantibodies (MAs) in the sera of 788 patients with suspected MSA and confirmed Sars-CoV-2 infection and/or vaccination from 11 Spanish hospitals in 2022, revealing an anti-MDA5+ prevalence of 11.66%. Anti-MDA5+ was a criterion for inclusion in the MSA non-ARS (non-anti-aminoacyl tRNA synthetase) group, which was the most numerous and the one with the highest number of patients reporting Sars-CoV-2 infection and vaccination with non-mRNA vaccines. In addition, anti-MDA5 was detected at low concentrations (LPOS) associated with lung involvement, given its binding to RP-ILD. An increase in serum titers of anti-MDA5 AABs was also reported in patients who developed neuro post-infectious sequelae after SARS-CoV-2 infection (neuro-PASC or NP), symptoms of which include brain fog, headache, myalgia, and fatigue (25). NP was common in patients with mild acute COVID-19 infection (94.1%), along with significant levels of anti-MDA5 antibodies, which showed a striking correlation with mild primary infections rather than breakthrough (post-vaccination) infections. This suggests the protective role of vaccination against SARS-CoV-2 and its capability of inducing autoimmunity. In fact, vaccination has been found to decouple antiviral immunity against SARS-CoV-2 from humoral autoimmunity (26). COVID-19 autoimmunity has been found to follow paradoxical sex-specific patterns (27): conventionally, females are known to be more susceptible than males to autoimmune conditions, but it has been observed that in mild symptomatic SARS-CoV-2 infections the auto-antibody response is more pronounced in males, while in females the AAB burden is relevant after asymptomatic infections (27). Among the AABs measured, anti-MDA5 synthesis was triggered at the beginning of infection and correlated positively with symptoms severity (27).

### MDA5+ DM development after COVID-19 disease

A retrospective study (19) conducted in Yorkshire (UK) reported a surge in MDA5+ DM cases during the COVID-19 pandemic between 2020 and 2022. The peak of MDA5+ rates occurred in 2021 and overlapped with SARS-CoV-2 infections in the region. This is the largest study conducted to describe the characteristics and outcome of this syndrome in 2021 (19). Approximately 42% of the cases discussed presented with progressive ILD, the typical aggressive course of MDA5+ ILD. Epidemiological and transcriptomic observations suggest that the increased incidence of autoimmunity from MDA5+ and ILD that occurred simultaneously during COVID-19 could be related to an aberrant IFN type 1 - centric response. These observations lead to the proposal of the term “MDA5+ autoimmunity and Interstitial Pneumonitis Contemporaneous with COVID-19 pandemic” (MIP-C) (19). This acronym is ascribed to the distinct features from classic anti-MDA5+ DM, including the Caucasian-predominant population instead of the historically reported East Asian, as well as the lower rate of ILD evident in 42% of cases compared to what has historically been reported in MDA5+ DM (19). An observational survey about MDA5+ DM was reviewed by John Hopkins Institutional Review Board and conducted in the USA between December 2022 and January 2023, using a Google anonymous format. Among the 46 participants who responded, 44% reported a Sars-CoV-2 infection before the onset of symptoms, with a quarter of these subjects being positive to anti-MDA5 after the infection. Of responders, 20% declared subsequent prolonged symptoms, which were associated with long-COVID. The 14% reported no vaccination against Sars-CoV-2 (28). A Chinese retrospective study from 2023 (29) analyzed the onset timing of RP-ILD and mortality in a cohort of 272 patients with MDA5+ DM. They found that the window of risk is just three months, with 50% RP-ILD and 46% mortality. Furthermore, the authors speculated that the rapid onset of RP-ILD may be triggered by viral infections such as Sars-CoV-2, considering that it is an autoimmune symptom shared by both MDA5+ DM and COVID-19. This is also attested by the finding of anti-MDA5 autoantibodies in the serum of COVID-19 patients, implying a possible common etiopathology with MDA5+ DM. Cardiac involvement in IIM is a phenomenon reported in the literature, whereas it is rarer in MDA5+ DM (30). In a 2022 retrospective study (31), the authors described an abnormality in the electrocardiographic tracing, identifiable as a T wave with decreased amplitude and associate it with the presence of anti-MDA5 auto-antibodies. This results in a subclinical abnormality of ventricular conduction during the systole-diastole transition. Again, the authors trace such a phenomenon to concomitant viral infections, particularly by viruses such as Sars-CoV-2. The pediatric DM variant is known as juvenile idiopathic inflammatory myositis (JIIM). A recent work (32) reported diagnosis of JIIM in 25% of the study cohort during the post-pandemic period, 77% of which had anti-MDA5 AABs after exposure to SARS-CoV-2. Although the study population was small, thereby reducing statistical power, the patients in this study tended to be older than pre-pandemic patients, female, and with non-specific cutaneous manifestations (32).

### Molecular signature of type-I interferonopathy and pathogenetic mechanisms

MDA5 is a specific cytoplasmic sensor for viral dsRNA intermediates synthesized after infection, as it is required to counteract Sars-CoV-2 presence (33). Binding to its target activates transcription of IFN-I and INF-III genes, resulting in synthesis of IFN-β and IFN-γ, both of which are essential for antiviral innate immunity (33, 34). The IFN pathway is overactive in autoimmune diseases such as DM, thereby facilitating presentation of antigens to aberrant extrafollicular B lymphocyte clones (2, 4). On a molecular level, Song et al. found in 2021 that unanchored chains of K63 polyubiquitin are required to stabilize interactions by tethering between the CARD domains of MDA5 and mitochondrial antiviral signaling (MAVS) proteins to call up TBK1-IRF3 factors, which are necessary to trigger transcription of IFN-I and -III genes (35). Ubiquitin-like proteins, such as ISG15, covalently modify the CARD domains of RIG-I and probably do the same with MDA5. Sars-CoV-2 is able to disrupt such interactions by its papain-like proteases, resulting in non-transcription of IFN-I genes, therefore antagonizing MDA5 activity (35). Thang et al. (36) in the same year found that ubiquitin-specific protease 8 (USP8) inactivation brings to MDA5 proteasome degradation, suppressing antiviral activity and autoimmunity. In fact, viral infections boost USP8 activation via AKT-dependent phosphorylation, increasing MDA5 expression. The authors conclude that USP8 inhibition could provide potential treatment for autoimmune diseases, by redirecting MDA5 to degradation and, in turn, inhibiting overactive type-I and -III IFN activation (36). Such an increase of MDA5 after viral infections was studied by Ghoreshi et al. in 2024 (37), who measured the expression level of the cellular viral RNA sensors in COVID-19 patients, particularly in their peripheral blood mononuclear cells (PBMCs) and in nasopharyngeal epithelial cells. They found a positive correlation between COVID-19 severity and MDA5 expression in the respiratory tract, coherently with the activation of the IFN-I pathways to counteract the Sars-CoV-2 presence (37). The IFN-I signature, and its correlation with anti-MDA5 antibody levels, has been investigated by Wang Y. et al, analyzing the transcriptomic profiles of PBMCs in individuals affected by MDA5+ DM using RNA-seq (38). The analyses revealed an enriched population of differentially expressed mRNAs, particularly mRNAs encoding antiviral and cytokine-related genes, in patients with MDA5+ DM. Furthermore, the analyzed patient group exhibited higher expression of type-I IFN signature genes compared to the healthy donor group. Further analysis of the clinical parameters in MDA5+ DM patients revealed a significant correlation between the anti-MDA5+ titer and the type-I IFN score. A similar result was obtained by the work of Zhang Z. et al. (39), who used differential expression analysis to analyze the transcriptomic data of both COVID-19 and IIMs obtained from the GEO database. The research identified 86 genes in common expressed in the two diseases and classified them into two main clusters: inflammatory factors (including MAPK signaling) and interferon-mediated signaling, which are active during antiviral immunity and whose overexpression is known to lead to autoimmune diseases. Ichimura et al. in 2024 (40) described a mice model for MDA5+ DM. They found that MDA5+-immunized mice developed lungs inflammation with RP-ILD after treatment with poly (I:C) used to mimic viral infection. In addition, they found an increment of inflammatory cells, namely neutrophils, macrophages CD11b+, and CD4+ T cells, in the mice lung parenchyma from bronchioalveolar lavage fluid (BALF) and histological samples when compared with controls. ILD mice lungs present augmented collagen deposition compatible with tissue fibrosis. After RNA sequencing, differentially expressed genes (DEGs) were analyzed, resulting in augmented expression of type-I IFN-related genes, MHC MDA5+ DM PBMCs, finding type-I IFN overactivation of innate and adaptive immune cells (40). A similar work has been conducted by He et al. (41) in the same year, analyzing the PBMCs transcriptome with scRNA-seq on eight MDA5+DM patients. Particularly, they recognized a different subtype of CD14+ monocytes expressing IFIH1 (MDA5) and IFI27 (ISG12). IFI27 has been recognized as a biomarker for early Sars-CoV-2 infection, corroborating the association with viral infections. The authors concluded that IFI27 expression persists even after the acute response however, it isn’t clear if CD14+ monocytes are effectively the MDA5 antigens’ source (41). In fact, MDA5 has been found not only in cytoplasm, but also in secretory vesicles and cell surface cell membrane of neutrophils (42) and its expression could be enhanced with a positive feedback loop during a viral infection. A link between monocytes, which play a role in the pathogenesis of anti-MDA5+ RP-ILD and COVID-19 has been investigated by Gono et al. (20); therefore, an analysis on the association between miRNA and mRNA was conducted on peripheral blood monocytes extracted from three MDA5+ DM patients and compared with the results from three healthy controls. The gene interaction network was evaluated by Ingenuity Pathway Analysis, which revealed information regarding miRNA targeting and identified an antiviral proinflammatory network, like that against SARS-CoV-2, orchestrated by activated macrophages (20). In 2025, using scRNA-seq, Shi et al. (43) compared the transcriptomic profile between pulmonary cells from BALF and the PBMCs, obtained by MDA5+ DM patients with RP-ILD. The analysis revealed differences in the pulmonary and the peripheral blood background such that peripheral monocytes are characterized by an immunosuppressive phenotype, with reduced cytokine and MHC type-II expression, and are recruited in the lungs where they differentiate in monocyte-derived alveolar macrophages (Mo-AMs). The Mo-AMs establish a proinflammatory state in lungs, attracting other innate and adaptive immune cells, including natural killer cells (NK), CD4+, CD8+ T cells and B cells from the periphery and causing lymphopenia. Transcriptomic analysis on peripheral monocytes revealed, again, the expression of antiviral IFN I and IFN-γ pathways in order to be recruited in the inflammation site (43).

### Rheumatological treatment approaches

COVID-19 shares many similarities with MDA5+ DM, including cytokine storm triggered by the hyperinflammatory (HI) phenotype, which leads to development of AABs such as anti-MDA5 in genetically predisposed individuals (19, 20). This pathophysiological process has led to development of pharmaceutical molecules such as Baricitinib, which prevents severe viral disease by modulating the patient’s immune phenotype (44). Baricitinib has been studied in combination with a 6-methylpredinosolone pulse regimen in COVID-19 patients hospitalized in the intensive care unit (ICU). This therapeutic regimen resulted in a significant reduction in mortality, as well as in expression of inflammatory markers (45). Baricitinib acts by modulating the Janus kinase (JAK1/2), which is activated through the IFN-I pathway during RNA virus infection, and whose involvement in development of MDA5+ DM is widely accepted. Replication of viral RNA triggers synthesis of IFN-I class molecules (IFN-β and IFN-λ) by infected cells, and this signature has emerged as a potential risk factor for the pathogenesis of MDA5+ DM after SARS-CoV-2 infection (22, 34, 46). There is also limited evidence that Baricitinib can be effective in patients with RP-ILD secondary to IIM (47), and it is postulated to be of even greater benefit than Tofacitinib, another Janus kinase inhibitor (45).

### Case reports: post-COVID manifestations of MDA5+ DM

Our research described ten case reports, everyone with clinical onset of MDA5+ DM during COVID-19 or 1–3 months to 1 year after its resolution. In every case presented herein, patients reported at least one or more of the following symptoms: arthralgia, weakness, Gottron’s papules, heliotrope rash, periorbital edema, dyspnea and pneumonitis with CT bilateral ground-glass opacities (or RP-ILD) (**Table 1**). Laboratory testing, when reported, showed ferritin, C-reactive protein (CRP) and/or CK over the limits, together with leukopenia and/or lymphopenia. Serological analysis revealed anti-MDA5 antibodies, confirming, along with the clinical signs, a diagnosis of MDA5+ DM. In 2021, Keshtkarjahromi et al. (48) reported a case of a 65-years-old Caucasian female with a history of psoriasis and recent COVID-19 diagnosis. The patient presented with generalized weakness, arthralgia, heliotrope rash, erythematous rash, Gottron’s papules, shortness of breath, weight loss and bilateral pulmonary infiltrations. Despite pharmacological treatment, she developed macrophage activation syndrome (MAS), with progressive clinical worsening and exitus. In 2022, Anderle et al. (16) reported the case of a 20-year-old Bangladeshi boy who developed MDA5+ DM with RP-ILD, which further progressed to acute respiratory distress syndrome (ARDS), requiring an urgent lung transplantation. In the same year, Juganaru et al. (49) described the case of a 31-year-old woman developing skin rash and arthralgia shortly after resulting positive to COVID-19, while Suparna et al. (50) reported cases of skin rash and arthralgia in two women aged 66 and 54 years, respectively, three months after resolution of Sars-CoV-2 infection. Only the older one developed anti-MDA5 AABs, that were not detected in the latter. In 2023, Stępień et al. (51) reported the onset of cutaneous ulcerations and Raynaud’s phenomenon in a 57-year-old woman, with calcium deposition in the lower limbs (calcinosis cutis). In the same year Man Ng and Ho Leung (52) described skin rash with RP-ILD development within days after COVID-19 resolution. In 2024 Lau et al. (53) reported RP-ILD with anti-MDA5 AABs positivity in a two-year-old baby, with fatal consequences; Arai et al. (54) described a multiple AABs positivity after Sars-CoV-2 infection, specifically anti-MDA5, anti-ARS and MPO-ANCA, while Armstrong et al. (55) and Puthumana et al. (56) presented two and three cases, respectively, characterized by Slow-Progressive ILD with anti-MDA5 AABs in their post-COVID period. As for vaccination, only two female patients, described in two different case reports, were previously vaccinated for COVID-19 (50, 52). One patient completed the three-doses course of BioNTech mRNA vaccination, while the other reported no SARS-CoV-2 infection and received a second dose of Covishield (viral vector-based vaccine), but was serologically negative for anti-MDA5 and only positive for anti-SAE1. None of the patients presented in the case reports exhibited prior autoimmune diseases, except for the one with psoriasis reported from Keshtkarjaromi et al. (48). The most prescribed drugs in these reports were corticosteroids, Rituximab, cyclophosphamide, mycophenolate, and antibiotics. Only in one case Baricitinib was administered (52).

## Discussion

### Main findings

The aim of the present review was to assess the role of SARS-CoV-2 in the development of a rheumatic condition defined during the 239^th^ ENMC international workshop as MDA5+ dermatomyositis (10). In 2023, Tesch et al. assessed the risk of developing an autoimmune disease in patients with a previous COVID-19 diagnosis (Tesch et al). Compared to the control group (people without COVID-19), subjects with previous COVID-19 had a 43% higher likelihood of developing an autoimmune disease, whereas subjects with previous COVID-19 and any pre-existing autoimmune disease had a 23% higher risk of developing another autoimmune disease (57). Patients affected by interstitial inflammatory myositis (IIM) report symmetrical proximal muscle inflammation, arthralgia, weakness and extra-muscular manifestations such as skin rash. It is known that each IIM subtype is characterized by serum positivity to a specific AAB, reflecting a subset of clinical manifestations (8). Typically, anti-TIF1-γ DM occurs predominantly with skin inflammation, as well as anti-SAE DM, whereas anti-SRP myositis manifests predominantly with muscle weakness and necrotizing myopathy (8). MDA5+ DM is a subtype of IIM with little to no muscular involvement (amyopathic) (7), which commonly worsens in RP-ILD and was first described in Japan. It is unclear if the muscular involvement depends on ethnicity (10). It was regarded under the umbrella of CADM because affected patients were either hypomyopathic or amyopathic (15). The disease name derives from the presence of AABs first recognized by Sato et al. in 2009, specific to MDA5 (9, 10), a cytosolic sensor for viral RNA, which is a member of the RIG-I like family receptors (RLRs) encoded by the *IFIH1* gene, the function of which is to bind dsRNA intermediates during virus replication, triggering expression of IFN-I and IFN-III genes, such as IFN-β and IFN-λ. This pathway activates the innate immune response in lung epithelial cells during SARS-CoV-2 infection (17, 34). After dsRNA binding, unanchored K63 poly-ubiquitin long chains are required to stabilize by tethering the interaction between MDA5 CARD domains and the MAVS, which are necessary to recruit TBK1-IRF3 factors for IFN-I and -III genes transcription. Sars-CoV-2 is thought to perturb such interactions thanks to its papain-like protease, and by antagonizing MDA5 activity (35). Viral infections indirectly prevent MDA5 proteasome-degradation by activating USP8 via AKT-dependent. Specific IFIH1 variants results in MDA5+ overactivation, causing autoimmune diseases like Aicardi-Goutières syndrome (AGS), Singleton-Meyer syndrome (SMS), dermatomyositis and type-1 diabetes. USP8 inhibition allows MDA5 ubiquitination and proteolysis, preventing its overactivation and providing a potential target to impair autoimmunity development (36). MDA5+ DM clinical manifestations can be clustered in three main subgroups: anti-MDA5+ rheumatic DM with skin lesions and/or arthralgia/arthritis; anti-MDA5+ vasculopathic DM, characterized by skin vasculopathy and Raynaud’s phenomenon; anti-MDA5+ RP-ILD DM, with a prevalence of 50-100%, characterized by poor prognosis and high early mortality (8, 11). Some patients also presented slight ECG anomalies, in particular a wide T wave, suggesting a sub-clinical myocardial damage impairing ventricular repolarization (31). This wide clinical spectrum led to thinking of MDA5+ DM as a systemic syndrome, rather than a mere muscular autoimmune disease. Involvement of SARS-CoV-2 in development of MDA5+ DM is associated with an overexpression of IFN-I signature genes in MDA5+ patients, which correlate positively with the titer of anti-MDA5 antibodies (18). The studies collected in the present review highlight the strong role of the IFN-I pathways overactivation in the MDA5+ DM monocytes: scRNAseq analysis revealed IFN-I molecular signature and the activation of genes related to antiviral immunity. Particularly, peripheral circulating monocytes are recruited in lungs, where they transit from immunosuppressive phenotype to proinflammatory and profibrotic monocyte-derived alveolar macrophages (Mo-AMs). The secreted chemokine and cytokine subsequently attract innate and adaptive immune cells in lung parenchyma from peripheral circulation, causing leukopenia and/or lymphopenia observed in patients (43). Noteworthy, in MDA5+ DM patients a specific subtype of CD14^+^ monocytes strongly expressing IFI27, a biomarker reported for mild early Sars-CoV-2 infection, has been discovered (41, 58). The authors reported the same IFN-I overactivation in the adaptive B and T cells, in line with another study (59). Also, analysis of miRNA-mRNA association in circulating monocytes revealed an IFN-I proinflammatory network orchestrated by monocytes, which are activated by viral RNA acting as an upstream signal. This could result in MDA5+ DM and, consequently, RP-ILD (20). Analysis of transcriptome data obtained from 44 COVID-19 and 31 IIM patients revealed the transcription of 91 genes in common, including those implicated in type-I IFN cellular response. After application of machine learning methods, three key genes have been recognized: CDKN1A, STAB1 and, again IFI27. Another proof for the viral-trigger model comes from Ichimura et al. work, who used poly (I:C) to mimic a viral infection in a mice model previously immunized against MDA5. The mice developed ILD with consequent fibrosis, and transcriptomic analysis revealed activation of IFN-I genes, MHC type II on the bronchiolar epithelium and IL-6. Based on these assumptions, another study examined involvement of IFN-I cellular pathways in COVID-19 by analyzing the efficacy of recent pharmaceutical drugs such as Baricitinib, which exerts its activity by inhibiting JAK, in the JAK/STAT complex signaling pathway, which is upregulated in rheumatic conditions, with aberrant activation (46). Treatment with the Baricitinib plus corticosteroids regimen in COVID-19 patients with a hyperinflammatory phenotype (HI) and admitted to ICU had better outcomes, including a significant reduction in mortality, compared to classic treatment (i.e., corticosteroids plus Remdesivir) (45). The efficacy of Baricitinib plus corticosteroids, along with transcriptomic studies of IFN-I and the discovery of anti-MDA5 AABs in serum from COVID-19 patients, confirms that SARS-CoV-2 can trigger HI in genetically predisposed patients, which can evolve into autoimmune disorders such as MDA5+ DM (20). Such IFN-centric inflammatory responses are thought to be the cause of the increase in MDA5 positivity tests (+ 4,8%), the rise of ground-glass opacities and pulmonary fibrosis in ILD patients, and the increase of cutaneous manifestations, Raynaud’s phenomena and proximal myopathy in the non-ILD patients observed during the peak in COVID-19 cases in 2021 in Yorkshire, UK. In this study, 2/3 of population developed a condition which the authors defined “MDA5-autoimmunity and interstitial pneumonitis contemporaneous with the COVID-19 pandemic” (i.e., MIP-C) (19). This phenotypic variant shows some epidemiological and clinical differences from classic MDA5+ DM; for example, the affected population was Caucasian rather than the more typical East Asian, and rates of ILD were lower than historically reported for MDA5+ DM. The increase of serum anti-MDA5 AABs in patients overlapped with the peak vaccination period, suggesting that development of herd immunity led to milder COVID-19 infections, thereby favoring emergence of MIP-C. Furthermore, *IFIH1* variant rs1990760 (Ala946Thr) appears to confer age-dependent protection against cytokine storm and autoinflammation (19). The development of MDA5+ DM after Sars-CoV-2 infection has also been proven by a recent survey in USA and reviewed by John Hopkins Institutional Review Board, where 44% of participants reported onset of the autoimmune illness after Sars-CoV-2 infection. A slight post-pandemic increase in anti-MDA5+ juvenile interstitial inflammatory dermatomyositis (i.e., JIIM) was observed at the Children’s Hospital of Montefiore (NY). Although the data was not statistically significant, post-pandemic patients were older and female, with non-specific cutaneous manifestations, consistently with results reported in a previous study (32, 60). The concentration of anti-MDA5 in serum correlates with emergence of RP-ILD, which presents clinically as ground-glass opacities on chest radiology and occurs typically within 6–12 months post-diagnosis (11, 29, 61, 62). MDA5+ DM shares many features with COVID-19, including chest radiology, cytokine storm, and even treatment approaches (corticosteroids and Janus Kinase inhibitors), suggesting a common pathophysiological mechanism (18). The development of anti-MDA5 AABs begins during Sars-CoV-2 infection: G. Wang et al. reported in 2021 that AABs targeting MDA5 have been observed not only in those with MDA5+ DM, but also in the serum of COVID-19 patients (18). They found a positive correlation between the AABs titer, COVID-19 severity, and adverse outcomes such that the higher the AAB levels, the more COVID-19 symptoms were severe, and the lower the survival rates. Anti-MDA5 AABs were detected in nearly half (48.2%) of COVID-19 patients in the study, and it is speculated that their development in genetically predisposed individuals may be triggered by uncontrolled autoinflammation and autoimmune responses during the acute phase of infection. Evidence suggests that in a significant proportion of COVID-19 patients these AABs are generated by immune responses against structural and non-structural viral protein epitopes that induce molecular mimicry (22). In fact, three immunologic epitopes with high sequence similarity to SARS-CoV-2 have been identified in patients with autoimmune DM (63). Moreover, it is hypothesized that expression of MDA5 and the dsRNA-MDA5 complex could be upregulated in tissues before being released by cell lysis, with concomitant antigen presentation and production of AABs (64). This correlates with the findings by Ghoreshi et al. (37), who observed an augmented expression of viral RNA sensors, including MDA5, positively correlating with infection’s early stages and severe stages, respectively, in the epithelial lung cells and in PBMCs. Furthermore, a non-canonical expression of MDA5 on neutrophils’ surface membrane has been observed, as well as in secretory vesicles, after a positive feedback loop during viral infections (42). It can be postulated that certain viruses, like Sars-CoV-2, could enhance the expression of MDA5 in specific cell types, providing the necessary autoantigens for autoimmunity. The anti-MDA5 AABs could be synthesized by B cells derived from the extrafollicular pathway which, unlike those maturing in germinal centers, lack certain maturation checkpoints that prevent autoreactivity (4). Patients with severe COVID-19 exhibit higher levels of extrafollicular B lymphocytes, which are prone to producing pathogenic AABs (4). Evidence suggests that milder as well as severe COVID-19 infections increase production of AABs and subsequent development of autoimmune diseases (25), particularly in patients who later experienced long COVID and/or neuro post-infectious sequelae of SARS-CoV-2 infection (i.e., neuro-PASC or NP). Growing evidence suggests that Long COVID seems to be an autoimmune disease in which the infected patient produces AABs resulting in a chronic, persistent inflammatory response (65). MDA5+ levels in those patients showed a strong correlation with a primary but not a breakthrough (post-vaccination) SARS-CoV-2 infection, suggesting that vaccination may help prevent MDA5+-driven autoimmune responses, as well as reducing the risk for developing other autoimmune diseases (25, 66). Although several cases reported hypothesized a causal role of COVID-19 vaccination in the onset of MDA5+ DM, many of the patients described in the case reports also reported mild COVID-19 prior to vaccine inoculation (67). In addition, Jaycox et al. (26) have recently shown that patients with previous COVID-19 present a characteristic pattern of new and increased AAB (including anti-MDA5), whereas vaccinated individuals do not present distinct autoimmune features. They also compared the presence of small AABs reactivities between vaccinated and unvaccinated cohorts, showing no differences and attributing these serological fluctuations to physiological variations rather than to the vaccination itself. AABs production, on the other hand, was linked to an exuberant B-lymphocyte response, rather than a dysregulated IFN-I pathway during viral inflammatory burden (26). This strengthens the safety profile of the mRNA vaccine and its ability to separate SARS-CoV-2 autoimmunity from the potential long-term autoimmune sequelae of COVID-19 (4, 18, 26, 34, 66). Most NP patients with primary infections were infected during the Delta wave (2021), while NP patients with breakthrough infections acquired SARS-CoV2 infection during the Omicron wave (2022) (25). This matches with the increase of MDA5 positivity reported in Yorkshire (UK), which peaked in 2021 during the Delta wave outbreak. Synthesis and persistence of anti-MDA5 AABs is sex-specific: AABs responses are more pronounced in women following asymptomatic illness and in men after a mild COVID-19 infection (27), confirming the typical autoimmune disease link between mild acute primary infection and a higher probability of developing AABs (25). SARS-CoV-2 constitutes an environmental trigger which could cause latent autoimmune disease through mechanisms like molecular mimicry, epitope spreading, and bystander activation (68, 69). This could enhance anti-MDA5 serum fluctuations preceding true positivity and, in genetically predisposed individuals, in the development of a frank rheumatic condition such as MDA5+ DM, which is observed as the MIP-C phenotype variant in Caucasian individuals (19). David P. et al. demonstrated a surge in new cases of anti-MDA5 however, other myositis-specific autoantibodies (MSAs) did not show the same pattern of increase (19). Interestingly, differences in the antibody profile were reported during the pandemic in different geographic groups (24), for instance, Asian populations showed a higher frequency of anti-MDA5 antibodies than Caucasian populations (24, 64). Anti-MDA5+ has been found to be present in low-positive (LPOS) serum concentrations in many patients (24). Although some studies suggest that LPOS should be interpreted with caution and many authors describe it as a possible false-positive clinical finding, it is important to interpret the results in relation to the patient’s clinical history. The results obtained by Garcìa-Bravo et al. demonstrated a higher frequency of anti-MDA5 (17.16%) with LPOS associated with pulmonary involvement (24). This statement could support the work of Wang G et al. on the presence of anti-MDA5 autoantibodies in the serum of individuals with SARS-CoV-2 infection (18).

These observations, along with a large spectrum of disease represented by ground glass opacities and pulmonary fibrosis in ILD patients, as well as skin rashes, Raynaud’s phenomena, vasculitis in non-ILD patients (19), suggest that the so-called anti-MDA5+DM is rather a systemic syndrome, defined as anti-MDA5 syndrome – AMD, rather than a proper musculocutaneous disease (11). However, the term myositis-specific antibody seems inappropriate, because many anti-MDA5+ patients are amyopathic (11). The MDA5 serum positivity concomitant with a lower rate of RP-ILD in Caucasian populations led to the MIP-C acronym to be coined in the context of AMD (19).

Case reports presented herein (**Table 1**) developed various clinical signs, such as arthralgia, weakness, skin rash, shortness of breath, ground glass opacities elevated serum ferritin levels and even a rare complication like calcinosis cutis, aligned mainly with the RP-ILD sub-phenotype and mirroring its 50-100% prevalence. The cases here presented developed symptoms during or after the resolution of COVID-19, in a female-dominant population of various ethnicities (16, 48–56). In only one case RP-ILD further degenerated to ARDS (16), requiring an urgent lung transplant. The case presented by Keshtkarjahromi et al. showed also a Macrophage Activation Syndrome (MAS), exacerbating the diagnosis of MDA5+ DM with lymphopenia and anemia (48). An interesting case is reported by Puthumana et al. and Armstrong et al. (55, 56), which showed in post-COVID patients the development of a slowly progressive ILD rather than a rapid one, confirming the wide phenotype spectrum of MDA5+DM. In every case here presented, elevated serum titers of anti-MDA5 were showed and confirmed during the infection or after its resolution.

### Limitations

This review has some limitations. Firstly, the studies enrolled were heterogeneous and some had small population samples. Secondly, in every case, the COVID-19 diagnosis was confirmed, but the methods used were not always clarified, and it was not clear whether serum analysis was conducted. Thirdly, laboratory values (CK, ferritin, CRP and hemoglobin) were not always mentioned in case reports here listed.

### Conclusions

SARS-CoV-2, like any RNA virus, has the ability to induce cytokine storm and autoinflammation in predisposed subjects due to type-I interferonopathy. This leads to development of autoimmune conditions such as MDA5+ DM in post-COVID patients, especially those who experienced Long COVID or PASC. Epidemiological and clinical data suggest a strong association between SARS-CoV-2 infection, development of MDA5+ DM and anti-MDA5 AABs (synthesized by autoreactive B lymphocytes matured via the extrafollicular pathway). Future research should investigate not only the role of SARS-CoV-2 infections in the context of a genetic predisposition mediated by variants of IFN-related genes (such as *IFIH1*), but also the potential treatment based on activation of IFN-I pathways.

## Data Availability

The Data presented in the study are deposited in the figshare reository. The original contributions presented in the study are publicly available. This data can be found here: [10.6084/m9.figshare.29940965].
