# Supplementary material for: Anti-MDA5+ dermatomyositis following SARS-COV-2 infections: a systematic review
Source: Front Immunol. 2025 Sep 2;16:1565803. doi: 10.3389/fimmu.2025.1565803 (PMC12436398; doi:10.3389/fimmu.2025.1565803)
Supplement: Supplementary file 1 [file DataSheet1.pdf]

APPENDIX A: ELECTRONIC RESEARCH STRATEGY

The following appendix describes the electronic research strategies utilized to identify the included studies in the systematic review.

A.1 Consulted databases:

- Google Scholar
- PubMed
- Scopus
- ScienceDirect

A.2 Detailed research strategies

The following research strategies have been adapted for each database, taking into account specific syntaxes and controlled vocabularies. MeSH terms were not included.

A.2.1 PubMed

Results number before duplicate records removal: 19

Keyword String**:** (Sars-CoV-2) AND (Dermatomyositis) AND (anti-MDA5) AND (COVID-19)

Limits applied: [Language: English; Years: 2020-2025]

A.2.2 Google Scholar

Results number before duplicate records removal: 503

Keyword String: (Sars-CoV-2) AND (Dermatomyositis) AND (anti-MDA5) AND (COVID-19)

Limits applied: [Language: English; Years: 2020-2025]

A.2.3 Scopus

Results number before duplicate records removal: 24

Keyword String: (Sars-CoV-2) AND (Dermatomyositis) AND (anti-MDA5) AND (COVID-19)

Limits applied: [Language: English; Years: 2020-2025]

A.2.4 ScienceDirect

Results number before duplicate records removal: 24

Keyword String: (Sars-CoV-2) AND (Dermatomyositis) AND (anti-MDA5) AND (COVID-19)

Limits applied: [Language: English; Years: 2020-2025]

Total records number before duplicates removal: 570

Total duplicates removed: 66

Records screened: 504

A.3 Motivations for Database Selection

The databases were selected for their broad coverage of biomedical and scientific literature, ensuring a comprehensive and multidisciplinary search on the topics of interest. PubMed and Scopus were chosen for their extensive indexing of medical and scientific articles, ScienceDirect for its specialization in scientific and technical publications, and Google Scholar for its ability to identify grey literature and articles not indexed in other databases.

A.4 Reference Management Software

The following software was used for bibliographic reference management and duplicate removal:

- JabRef (References management)
- Rayyan (duplicates removal and records screening)
